# The Ropivacaine Concentration Required for Ultrasound-Guided Rectus Sheath Block in Pediatric Patients Undergoing Single-Incision Laparoscopic Hernia Repair: A Sequential Allocation Dose-Finding Study

**DOI:** 10.7759/cureus.40668

**Published:** 2023-06-19

**Authors:** Keitaro Tachi, Shinichi Inomata, Makoto Tanaka

**Affiliations:** 1 Department of Anesthesiology, University of Tsukuba Hospital, Tsukuba, JPN; 2 Department of Anesthesiology, Institute of Clinical Medicine, University of Tsukuba, Tsukuba, JPN

**Keywords:** pediatric patient, effective concentration, ropivacaine, single-incision laparoscopic hernia repair, ultrasound-guided rectus sheath block

## Abstract

Background: The local anesthetic concentration required for ultrasound-guided rectus sheath block (RSB) in children remains unknown. Knowledge of appropriate ropivacaine concentration can help clinicians reduce local anesthetic toxicity risk when performing ultrasound-guided RSB in children. This study aimed to determine the appropriate ropivacaine concentration for ultrasound-guided RSB in children undergoing laparoscopic inguinal hernia repair.

Methods: In this single-arm prospective study with an up-down sequential allocation design of binary response variables, 18 consecutive children aged 11 months to 7 years undergoing single-incision laparoscopic percutaneous extraperitoneal closure were assessed. Orotracheal intubation was performed without intravenous anesthesia or a neuromuscular relaxant. After intubation, ultrasound-guided RSB was performed with a ropivacaine dose of 0.30 ml/kg (0.15 ml/kg per side). Dixon's up-and-down method was used to determine the concentration, starting from 0.25% in 0.05% increments. Surgery commenced ≥15 min following RSB. Body movement or a 20% increase in heart rate or systolic blood pressure within 1 min of surgery initiation determined an unsuccessful RSB. The 95% effective concentration of ropivacaine needed for successful RSB was calculated using the probit test.

Results: The 95% effective concentration of ropivacaine needed for successful ultrasound-guided RSB was 0.31% (95% confidence interval, 0.25-7.29). The highest concentration of ropivacaine required for successful ultrasound-guided RSB in the group of patients in this study was 0.3%.

Conclusion: The 95% effective concentration of ropivacaine (0.30 ml/kg total, 0.15 ml/kg per side) for ultrasound-guided RSB was 0.31% in children undergoing single-incision laparoscopic surgery under general anesthesia.

## Introduction

Rectus sheath block (RSB) is effective for analgesia in patients with umbilical or midline incision wounds. In recent years, there have been advances in performing rectus sheath blocks under ultrasound guidance. Ultrasound-guided RSB is reportedly more effective for analgesia than local anesthetic infiltration (LAI), following umbilical hernia surgery [1,2] and laparoscopic hernia repair [3] among pediatric patients. Compared to LAI, ultrasound-guided RSB accelerates the onset of anesthesia, prolongs its duration, and improves the postoperative pain score [1-3] but tends to increase the concentration of the local anesthetic in plasma [1,4]. Overdoses of local anesthetic due to regional anesthesia were reported in pediatric patients [5,6]. Thus, ultrasound-guided RSB should be performed with the smallest possible dose of a local anesthetic to avoid toxicity.

Prior research on the concentration of the local anesthetic required for ultrasound-guided RSB in children is lacking. The present prospective study determined the 95% effective concentration (EC95) of ropivacaine required at the time of skin incision for effective ultrasound-guided RSB in pediatric patients undergoing hernia repair by single-incision laparoscopic percutaneous extraperitoneal closure (SILPEC).

## Materials and methods

This single-armed prospective study with an up-down sequential allocation design of binary response variables was approved by the Institutional Ethical Committee of the University of Tsukuba Hospital (approval number: H24-047). The trial was registered in the University Hospital Medical Information Network Center (UMIN) clinical trials registry. The study was conducted in accordance with the principles of the Declaration of Helsinki. Patient enrolment started on August 1, 2016, and continued till July 31, 2019. Written informed consent was obtained from the study participants’ parents.

Eighteen consecutive children (aged 11 months to 7 years) with an American Society of Anesthesiologists (ASA) physical status of I-II who were undergoing SILPEC were included in the study. The exclusion criteria were as follows: ASA physical status III or higher, a contraindication to RSB, allergy to any drug used in anesthesia, an unsuccessful nerve block procedure, or parental refusal.

All patients fasted for ≥6 hours preoperatively and did not drink for ≥2 hours before surgery. No premedication was administered. Following the induction of general anesthesia with 60% nitrous oxide and 5% sevoflurane in oxygen, an intravenous (IV) catheter was inserted. Electrocardiographic parameters, non-invasive blood pressure, and percutaneous oxygen saturation were monitored continuously. Orotracheal intubation was performed without IV anesthesia or a neuromuscular relaxant. Ultrasound-guided RSB was then performed using an ultrasonographic machine with a 6-13 MHz linear probe (S-nerve®, Sonosite, Inc. Bothwell, WA USA) and a 25-gauge, 22-mm needle (Plexufix®, B. Braun Aesculap Japan Co., Ltd., Tokyo, Japan). The procedure was performed using the in-plane technique. The tip of the needle was advanced to the posterior sheath of the rectus abdominis muscle. After checking for backflow, ropivacaine (Anapeine®, AstraZeneca Japan, Osaka, Japan) was injected. The total dose of ropivacaine was 0.30 ml/kg (0.15 ml/kg per side). The concentration of ropivacaine was determined by Dixon's up-and-down method starting from 0.25% in increments of 0.05%. All blocks were performed by residents with more than six months of experience in anesthesiology. The procedure was supervised by the anesthesiologist staff. The procedure was excluded from the study if the ultrasound image determined that the local anesthetic was not injected at the correct location. After completion of the block, general anesthesia was maintained with sevoflurane (expiratory concentration, 2%) in oxygen and air (fraction of inspiratory oxygen, 0.4). All patients were ventilated artificially by a mechanical ventilator in pressure-controlled mode to maintain an end-tidal carbon dioxide concentration of 35 mmHg. An end-tidal nitrous oxide concentration was confirmed to be <3% at the start of surgery. The surgery was not commenced until ≥15 min after the completion of the RSB. If there was body movement or an increase in the patient’s heart rate or systolic blood pressure of ≥20% within 1 min of the start of the surgery, the RSB was deemed unsuccessful, and fentanyl 1-2 μg/kg was immediately injected. After the effect of the nerve block was determined, analgesia was administered as needed during the surgery.

With reference to a previous report [7], we continued to collect data until more than six pairs of data were obtained. The data are presented as the mean ± standard deviation (SD) as appropriate. We analyzed the ropivacaine concentration required for a successful block by using the probit regression model [7]. The data were processed using SAS System, version 6.12 (SAS Institute Inc., Cary, NC). To determine the concentration of ropivacaine required for a successful block in 95% of the children, the probability of success was assigned a value of 0.95. The EC95 with the 95% confidence interval (CI) was calculated.

## Results

**Figure 1:**
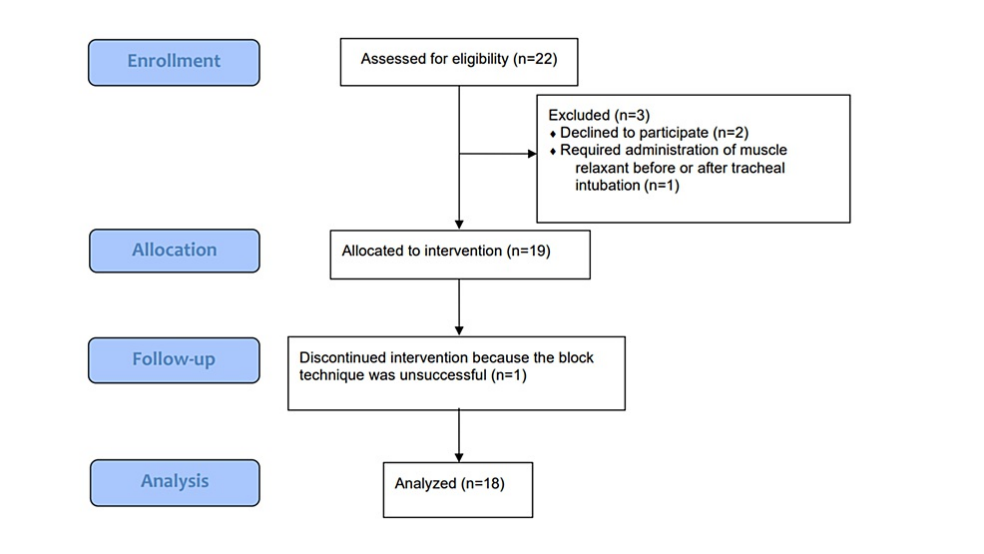
Flow chart showing patient recruitment.

Twenty-two children were enrolled in the study. Two children were excluded because we could not obtain consent from their parents; one child was excluded because the administration of a muscle relaxant was required before or after tracheal intubation, and another child was excluded because the block was determined to be unsuccessful in the ultrasound image, leaving 18 children for inclusion in the analysis (Figure 1).

The mean patient age was 59 ± 24 months. Seven patients were male, and 11 were female. The patient’s mean height was 103 ± 15 cm, and the patient’s mean weight was 17.2 ± 5.5 kg (Table 1).

**Table 1:** Demographic and clinical characteristics of paediatric patients The data are shown as the mean and SD or the number as appropriate. ASA-PS, American Society of Anesthesiologists physical status

Male/Female, n	7/11
Age, months	59 ± 24
Height, cm	103 ± 115
Weight, kg	17.2 ± 5.5
ASA-PS Ⅰ/Ⅱ	15/3
Anesthesia time, min	136 ± 16
Operating time, min	62 ± 12

**Figure 2:**
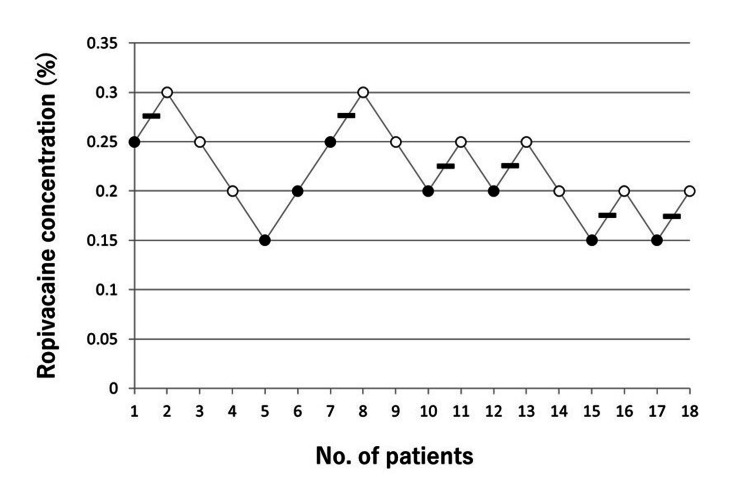
Sequential response of 18 children to initial incision with the up-and-down method. ●, failed block; ○, complete block; –,mid-point of “failed -complete” pair.

**Figure 3:**
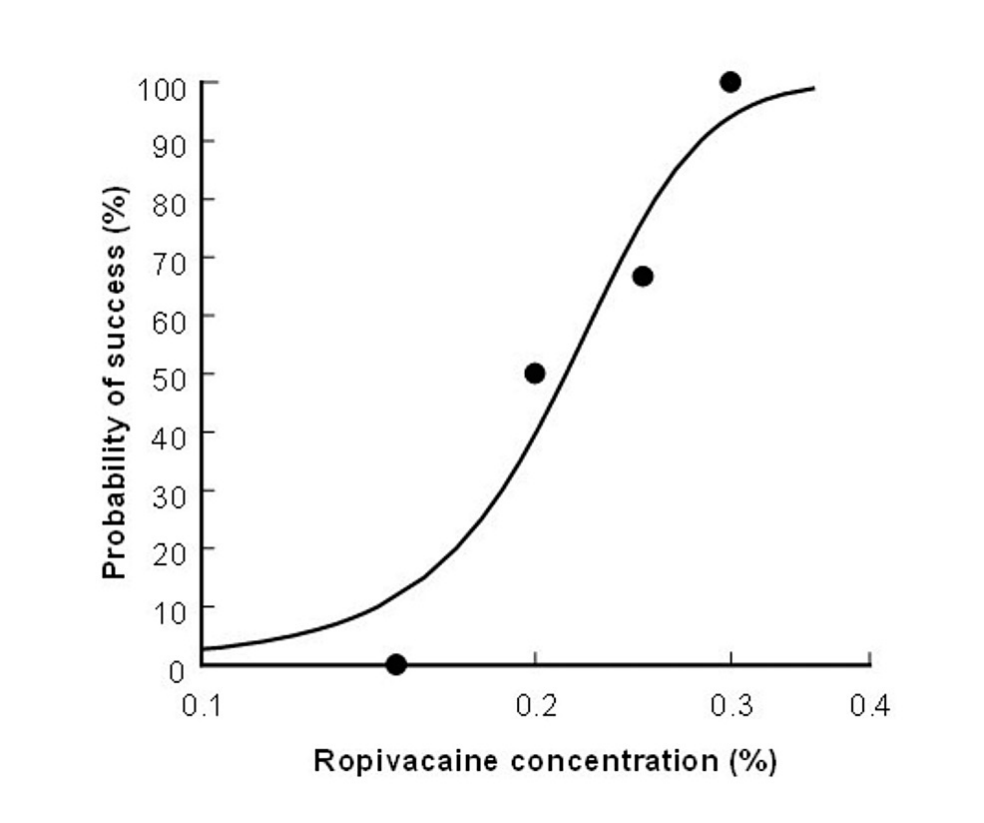
Dose-response curve for ropivacaine plotted using probit analysis. The 95% effective concentration was 0.31% (95% CI: 0.25-7.26)

The EC95 of ropivacaine required for successful RSB was 0.31% (95% CI 0.25-7.29) (Figures 2, 3).

The highest concentration of ropivacaine required for rectus sheath block in the group of patients in this study was 0.3% (Figure 2, Table 2).

**Table 2:** Percentage of patients who underwent a complete ultrasound-guided rectus sheath block

Ropivacaine concentration (%)	Success rate
0.15	0% (0/3)
0.2	57% (4/7)
0.25	80% (4/5)
0.3	100% (2/2)

## Discussion

This is the first study to determine the EC95 of ropivacaine when used for ultrasound-guided RSB in pediatric patients undergoing SILPEC. The results of our analyses revealed that the EC95 of ropivacaine for these patients was 0.31% (95% CI: 0.25-7.29). Laparoscopic surgery is now preferred to open surgery because it is associated with negligible scarring. More recently, laparoscopic surgery for an inguinal hernia that requires only a single incision in the umbilical cord has become popular because the wound is even more inconspicuous [8]. There is limited literature on postoperative pain after SILPEC in pediatric patients. The intensity of postoperative pain after single-incision laparoscopic surgery in pediatric patients remains controversial. In adults, single-incision laparoscopic surgery for inguinal hernia repair reportedly requires as much postoperative analgesia as three-port laparoscopic surgery [9]. Studies report that postoperative pain in children is more severe after laparoscopic hernia repair compared to open hernia repair [10,11]. Therefore, postoperative pain after SILPEC is likely to be as severe as that after a conventional method or three-port laparoscopic surgery and will require effective management.

The landmark-based RSB has been reported to be effective for analgesia in children undergoing umbilical hernia repair surgery [12] but has been associated with complications, including injury to the inferior epigastric vessels, intra-abdominal puncture, and retroperitoneal hematoma [13]. The ultrasound-guided RSB has been refined in recent years. The use of an ultrasound-guided nerve block allows the real-time imaging of the anatomic structure and the tip of the puncture needle, reducing the risk of intraneural, intravascular, or intraperitoneal injection. Furthermore, ultrasound-guided blocks were reported to have a higher success rate [14]. Ultrasound-guided RSB is also reported to provide effective analgesia after laparoscopic hernia repair in children [3]. Infiltration anesthesia at the port site is often used with an abdominal nerve block and is usually performed at the end of the surgical procedure but interferes with the surgical site. The advantage of ultrasound-guided RSB is that, unlike infiltration anesthesia, it can be performed before surgery without affecting the wound site.

Postoperative opioid-related side effects should be minimized to allow an uncomplicated postoperative recovery. Preoperative analgesia with RSB can reduce the intraoperative opioid requirement and subsequent side effects. In a study that compared the analgesic efficacy of preoperative RSB with that of infiltration anesthesia after surgery for an umbilical hernia, intraoperative opioid use was significantly reduced in the RSB group [2].

Ropivacaine was reported to reach its peak plasma concentration 45 min after ultrasound-guided RSB in adults [4]. Similar results have been reported for ultrasound-guided RSB with bupivacaine in pediatric patients [1]. Ropivacaine at a concentration of 0.75% has been associated with mild central neurotoxicity even when injected correctly in 10-ml unilateral doses for RSB in adult patients [4]. Overdose of ropivacaine for children has been reported [5,6]. Therefore, in pediatric patients, it is important to perform the RSB at the lowest effective dose to avoid toxicity.

The appropriate doses of local anesthetics required for ultrasound-guided RSB in children are yet to be determined. Therefore, in this study, we calculated the EC95 of ropivacaine at the time of the skin incision under ultrasound-guided RSB. The results of this study are consistent with previous reports of ultrasound-guided RSB for umbilical hernia and laparoscopic surgery in pediatric patients [15,16].

This study has some limitations. First, the study aimed to examine the analgesic effect of ultrasound-guided RSB at the time of skin incision. Therefore, postoperative pain was not evaluated. However, ultrasound-guided RSB has been previously reported to be at least as effective as LAI for postoperative analgesia after laparoscopic hernia repair in pediatric patients [3]. That study’s findings can be considered applicable to the concentration determined in the present study. Second, the analgesic assessments in this patient series were performed under 1 minimum alveolar concentration (MAC) [17] sevoflurane inhalation. The assessment of nerve block analgesia under conscious awareness in pediatric patients is difficult and dangerous because their cooperation cannot be obtained. From an ethical point of view, evaluation under inhalation of sevoflurane was considered appropriate.

## Conclusions

In summary, the EC95 of ropivacaine for ultrasound-guided RSB in this study of pediatric patients undergoing SILPEC under general anesthesia was 0.31% (95% CI: 0.25-7.29). In the realm of pediatric surgery, there is a growing trend towards single-port laparoscopic surgery at the umbilicus. To address the resulting pain from surgical wounds at this location, a multimodal analgesia approach is necessary. Therefore, ultrasound-guided RSB is expected to play a greater role in laparoscopic surgery for children. Knowledge of the EC95 of ropivacaine should reduce the risk of local anesthetic poisoning due to excessive administration when performing ultrasound-guided RSB in pediatric patients.
